# Approaches related to the effects of Covid-19 pandemics on financing of the healthcare system in Romania

**DOI:** 10.3389/fpubh.2022.940021

**Published:** 2022-07-27

**Authors:** Valentin Marian Antohi, Romeo Victor Ionescu, Monica Laura Zlati, Cristian Mirica

**Affiliations:** ^1^Department of Business Administration, Dunarea de Jos University, Galati, Romania; ^2^Department of Finance, Accounting and Economic Theory, Transylvania University, Brasov, Romania; ^3^Department of Administrative Sciences and Regional Studies, Dunarea de Jos University, Galati, Romania; ^4^Department of Accounting, Audit and Finance, Stefan cel Mare University, Suceava, Romania

**Keywords:** financing the medical system, pandemic stressors, strategic recovery measures, statistical dynamic model, sustainable financing

## Abstract

**Background:**

The healthcare financial system faced a significant disturbance of the budget balance after the outbreak of the pandemic, amid government measures to combat the disease. These measures have led to shifts in funding weights within the income and expenditure budget structure, with a focus on prevention and treatment of patients infected with SARS-COV 2. The purpose of this research is to analyse the financial balance of the healthcare system and the related modelling to support decision-makers in adopting and implementing appropriate financing measures for the pandemic.

**Methods:**

The working hypotheses of this study were tested using an econometric linear regression model based on the financing budgetary function, which matches funding to the specific need for each expenditure heading. SPSS 25 statistical software was used to define the model and to test the homogeneity of the data and their statistical relevance to the phenomenon under analysis.

**Results:**

The proposed model showed that there is a significant correlation of the dependent variable, Dynamics of budget revenues in the healthcare sector (TIM), with the regressors. We believe that a problem-solving rebalancing of allocations could help to eliminate the synergy in health. This redistribution should take into account the impact that economic and budgetary factors have on healthcare factors and vice versa, so that at some point after successive adjustments the minimum distance between forecast and realisation or between need and financing of need can be reached. The used data were analysed dynamically to assess changes in trend as absolute data do not allow the construction of an overall picture. Relative data captures changes in financing from year to year and can be linked to events such as pandemics, financial crises or inflation.

**Conclusions:**

In relation to the objectives of the research, it emerges that, under the impact of pandemic stress, measures to improve healthcare management, increase performance and streamline financial allocation are vulnerable and cannot counteract the effects that the pandemic has on the healthcare of the population as reflected in the morbidity and mortality indicators collected during the pandemic. In this regard, it is necessary a rethinking of the strategic healthcare management, a better planning of the procurement of medicines and healthcare supplies, a rethinking of the partnerships with the European Commission and other global entities. This approach can effectively improve the impact of the pandemic on the healthcare status of the population, a rebalancing of the demand-supply balance in healthcare and a maintenance of the strategic programmes, according to the objectives assumed in the planning, given that these programmes protect categories of people already medically affected.

## Background

The structure of the healthcare system in Romania is predominantly public, with a demographically shrinking population representing no more than 3.8% of the European population (1). In terms of healthcare indicators, Romania is below the European average in terms of birth expectancy (75.3 years vs. 80.9 years) and avoidable mortality ( 310 persons vs. 161 persons as Age-standardised mortality rates per 100 000 population) through healthcare insurance (1).

The main healthcare risk factors in the adult population in Romania are excessive episodic alcohol consumption, smoking and obesity. Spending on healthcare is the lowest in the EU, 1/3 of the EU average (1).

In Romania, social healthcare insurance is administered through public healthcare insurance houses (Bismarck model), situation that is also highlighted in the analysis carried out by some authors (2). Thus, the financing of healthcare care is provided from the budget of the Single National Health Insurance Fund (FNUASS), supplemented by amounts from the state budget and the state social insurance budget, as well as from the population's own income. The main public revenues for healthcare in Romania come from social healthcare insurance contributions, which are compulsory, as well as additional public revenues from subsidies from the state budget, the claw-back contribution and excise duties on tobacco and alcoholic beverages (“vice tax”) (3). In the case of private revenues for health, most financial resources come from direct payments, i.e., co-payments or charges for services. The financing of healthcare units and healthcare services is ensured by the National Health Insurance House (CNAS), and the county houses, from the Single National Health Insurance Fund (FNUASS), for the medical services provided to the population (3). Preventive activities, emergency services and National Health Programmes are financed by the Ministry of Health from the state budget allocated to the Ministry and from the Ministry's own revenues. Infrastructure expenditure is provided through the budgets of ministries and other central authorities for their own healthcare networks (4).

In this context, the financing of the healthcare system has gone through a continuous process of restructuring and improvement based on objectives proposed by the governing bodies. These objectives aimed at improving the management of the social healthcare insurance system, increasing the effectiveness of the National Health Insurance Fund (NHIF), improving access to healthcare services, increasing the coverage of services, improving the quality of medical care and legislative harmonisation in the healthcare segment in Romania (4).

These objectives were supported by concrete activities aimed at informing the insured through media channels, correlating with international best practice standards, improving the social image through communication and dissemination of information of broad interest in health. Internally, the focus was on strengthening the internal managerial control system, creating quality management structures in the healthcare units, moving to the process of the hospitals' accreditation and outpatient units, creating quality standards against which the medical act is currently assessed through the hospital accreditation process.

In this context, an important step was the regional healthcare restructuring. This phase has been partially implemented and has generated a transition period with certain vulnerabilities in the competence segment, and measures to classify accredited hospitals by competence levels and performance classes were necessary and implemented. So far there has been no success in implementing the regional hospital project, which would be of real help in the current pandemic context.

Through the hospital care provider payment system—DRG, tariffs for each diagnosis group (or relative values of tariffs) have been established, which are based on the adjacent costs of patients in each diagnosis group and the allocation of the hospital care budget to hospitals, based on the number and type of discharged patients (case-mix of each hospital) and the list of tariffs (or relative values) for each DRG. This funding system has not been in place since the outbreak of Covid-19, due to limited access to healthcare beneficiaries, with hospital healthcare providers being paid on a monthly out-of-pocket basis.

In the view of these aspects, we consider that a healthcare crisis may significantly affect the balance of healthcare system financing both through the need to impose immediate disease prevention measures and through the redistribution of the expenditure within the budget, leaving certain categories of expenditure intended to protect the healthcare of the insured vulnerable.

In this regard, we conducted a retrospective study analysing the financial balance of the healthcare system, resulting in some observations that allowed us to model the financing of the system according to the indicators of income and expenditure, highlighting the main causal indicators of funding imbalances. The modelling was carried out in two steps: comparing the 2010–2019 model with the 2010–2020 model, which revealed the disturbances resulting from the generalization of the pandemic in Romania.

As a result, we set the following critical issues as *research objectives*:

*O1:* Identifying budget revenue categories that react sensitively to pandemic risk.
*O2*: Identifying budget expenditure categories that react in a sensitive way to pandemic risk.
*O3:* Identifying expenditure categories that influence budget cash-flow variability and generate budget sensitivity under pandemic conditions.
*O4*: Quantifying the effects of the pandemic on long-term strategic objectives in health by forecasting the outcomes of the proposed model.

The article is of undeniable novelty, as it raises a highly topical and analysed issue (healthcare system financing in a pandemic context) on which no sufficiently strong causal relationships have been identified so far to redress the financial imbalances in health.

## Literature review

In the literature, we find elements that motivate proactive approaches to risk perception and concerns for healthcare system protection as a security factor.

In an interesting approach, the correlation between GDP, the population over 65 and the share of medical staff is studied by (5), who show that social expenditure on public healthcare depends directly proportional to the share of medical staff and inversely proportional to the number of people over 65, a population category with higher healthcare risk.

Another approach at EU level continues the above research, showing that there is a significant direct dependence between GDP/capita and healthcare expenditure/capita, but at the level of the population at significant risk, the influence on healthcare expenditure is small (6). The approach is supported by defining and testing an econometric model dedicated to correlating these indicators in a way that confirms that there are significant differences between EU countries, the authors giving the example of Romania and Bulgaria, which have the lowest levels of healthcare expenditure but the lowest healthcare status of the population in Europe.

An interesting approach to the health system in Romania by other authors (7), also focuses on legislative provisions and management instruments. Romanian hospitals have to face many challenges that affect their general and financial performance. As a result, a first step toward the recovery of the healthcare system in Romania, in the view of the authors of this study, is to achieve a sub-optimal hospital performance.

The analysis of the financing of the health care system in Ireland (8) shows that the private system requires additional expenditure that affects individual patient budgets. As a result, they can be divided into three categories according to the share of health expenditure in total capacity to pay.

Another comparative analysis (9) considers the Long-term indicator (LTC). While most states focus on the poor, Germany opted to make LTC universally. The German model has also been taken up by Japan, which offers great facilities for medical care now.

Some authors (10) have analysed the effects of the pandemic on the establishment of budgetary mechanisms in order to cover public healthcare expenditure in Romania. According to them, there is a differentiated regional development, the significant impact of which is polarized on the South and North East regions, where the hospital coverage rate is 45–50 units per region. At the other end of the scale are the South West and South East regions, which do not exceed a coverage rate of 35 hospitals per region. In terms of the OVID 19 impact on the economy, the authors show that, during the pandemic period, the increase in public debt directly influences GDP, forecasting a 37% increase in the indicator in 2022 compared to the beginning of the pandemic.

Other authors (11) analyse the pandemic crisis and its impact on the local finances, proposing a scheme to equalize the regional and local fiscal pressures to solve the problems generated by the development disparities.

The pandemic crisis has also had an effect on the healthcare promotion campaigns carried out by officials (12), so that there is an accumulation of impact factors that affect the economic and social balance and generate medical and social vulnerability across the population. The authors show that social responsibility and reorganisation can combat some of the effects of the pandemic crisis.

In a comparative analysis carried out by (13) it is shown that by comparing the psycho-social structure of Romanians and Italians, there is a higher preventive behaviour in Italy, which is reflected in the level of the adopted preventive measures. On the other hand, from the healthcare status point of view, at the date of the study (March 2020), Romanians show a higher optimism than Italians, considering the density curve projected by the authors. We appreciate that this optimism has triggered some of the current infection problems faced by the Romanian population and has indirectly increased the financial pressure of the pandemic on the healthcare budget.

According to (14), risk factors such as mortality caused by COVID 19 are analysed in relation to healthcare indicators (number of hospital beds per 1,000 inhabitants, public expenditure and age of the population affected by mortality), and it is found that, in a pandemic context, healthcare indicators are affected by more than 30%, and in the case of public expenditure on healthcare by more than 60% by the dynamics of the mortality rate of cases affected by COVID19.

From another perspective, the effect of the pandemic on the global economy and the implications for the Romanian economy have been analysed (15). The author points out that the most affected from a pandemic and economic point of view was the service sector, which includes healthcare services. For an economic recovery, it is important, in his opinion, to redefine the role of the international institutions and the relations with the international fora through assistance and financial support measures, including protectionist measures, which have long-term effects.

In his view (16), the impact of the pandemic on national economies is significant, peaking in the second quarter of 2020, coinciding with or preceding pandemic values.

In relation to the healthcare system in Romania, the strategies proposed by (17) are primarily aimed at promoting leadership, saving lives and reducing the economic impact. The authors identify the panel of vulnerabilities in healthcare as being related to poor infrastructure, lack of human resource experience, the influx of new staff into the system (fixed-term) and labour migration abroad. In an international analysis of the healthcare systems in terms of healthcare spending per capita, Romania ranks last, at least 10 times higher than spending in the US or 5 times higher than spending in Germany. This report is based on National Statistical Institute data, in which the average healthcare expenditure per capita in Romania is 1,115 USD, and the population ageing rate (+60) is 22.6% compared to 17% in the US or 28.2% in Germany. The authors conclude that, in the absence of sustained government intervention in the healthcare sector, the negative impact of Covid-19 is huge. The counter-weapons for rebuilding the healthcare system aim at forecasting costs, focusing on financing the private economy and entrepreneurship and providing the necessary mass of specialists in the healthcare sector.

A 17-year study of Eastern Europe shows that in most countries the value of social spending on healthcare is declining. Romania is in the middle of the ranking, with a negative accumulation rate of public expenditure on health, taxes and healthcare insurance. Of these, the most dynamically affected is healthcare expenditure, which fell by about 9% over the period. This aspect may be an attribute of the social healthcare system vulnerability in Romania, which has been faced with the successive reduction of some subsidized facilities from the state budget, being permanently restructured in order to cover the whole area of the medical solutions and services (18).

The analysis of the American healthcare system during the pandemic period shows some elements of vulnerability that can be transferred to other healthcare systems to make them more efficient. The authors of the research (19) show that monitoring the health status of patients, diagnosing and investigating public health and health hazards, informing, educating and engaging the population on health issues, mobilizing public-private partnerships to identify and solve community health problems, developing supportive public policies to improve the community health effort contribute to increasing the sustainable performance of the health system.

The impact of COVID-19 pandemic on the Finnish society in the view of some authors (20) was strong and difficult to anticipate. The government has adopted legislative measures to halt the spread of the virus, which have affected the economy, but not to the extent of the major developed countries. This is also due to the fact that Finland has opted for the “hybrid strategy” and has been able to accelerate the development of digital health services and telemedicine.

A comparative analysis of the American and global health system during the pandemic (21) shows that the R&D (Research & Development) component is paramount in addressing unanticipated risk, and that R&D issues can interfere with the whole range of services, from dignification, therapeutic regimens, mass replication of medical solutions, novel practice approaches, to financing procedures and administrative support of health care. According to the authors of this study, COVID−19 caused a financial impact of $3.86 trillion in 2020 global GDP. This means a major decrease of global GDP.

According to an EU-wide study (22), based on the share of individual costs in healthcare expenditure in the EU in 2013–2017, Romania ranks higher than Malta, Croatia and Bulgaria. The authors show that at the regional level, health spending has increased systematically over the period analysed with a significant level of disproportionality in allocations by country, with developed economies allowing much more substantial allocations than developing economies.

Some authors (4) highlight the fact that the medical system in Romania is strongly supported from public sources, the private contribution being around 20%. The authors make a forecast of the Social Health Insurance budget, which turns out to be on a strong upward trend. In some situations, such as a pandemic crisis, expenditure exceeds revenue, leading to budget destabilisation. Moreover, the authors also make a sectoral analysis of Romanian healthcare services, highlighting their different trends, among which that of Hospital Services and drugs that tend to monopolize the market.

An analysis of the effectiveness of European improved health systems COVID-19 (23) considers indicators such as: COVID-19 cases, physicians, nurses, hospital beds, health expenditure and COVID-19 deaths. From the analysis of these indicators, the authors explain that in the initial phase of the pandemic, European health systems were inefficient especially in Italy, Belgium, Spain and UK. In the second wave of the pandemic, these countries improved the performance of their health systems. Eastern European countries have not been able to sustain the performance of their health systems, as has happened in Bulgaria, Greece, Hungary and Romania. The pandemic lull may only be a transitional period and medical systems must be on high alert.

From the above study of the literature, accordingly with the other experts' opinions, it follows that the pandemic has a disruptive effect on the healthcare budget allocations, and the research has demonstrated the veracity of the assumptions underlying the research objectives. The following aspects were validated:

- The budgetary balance can be studied by analysing in a pandemic context revenue in relation to secondary healthcare expenditure.
- The structure of the financial allocation in a pandemic context undergoes substantive and formal changes under the impact of the population healthcare pressures.
- The structure of financial allocations per hospital undergoes changes in the pandemic context.
- Long-term strategic objectives tend to change and devalue under the impact of the pandemic.

## Methods

The authors proceeded to study the financing of the healthcare system in the period 2010-2020, collecting from the House National Health Insurance annual reports (24–34), information on budget execution, distribution of expenditure types in the income and expenditure budget, annual achievements by treatment and monitoring programme for different diseases, final point values in primary healthcare care and distribution of House National Health Insurance contracts for hospital services.

In order to achieve the aim of the research, to identify the influence of the COVID- 19 pandemic on the financing of the healthcare system in Romania, we defined, based on literature review and theoretical background, the following *working hypotheses:*

*H1:* The budgetary revenues from social assistance, under pandemic conditions, may be affected until the trend function changes in inverse proportion to budgetary revenues in the healthcare sector. This hypothesis is supported by a number of research studies (4, 15, 17, 24–34) and is in line with Research Objective 1.

*H2*: Spending on pharmaceuticals and specific healthcare supplies, including medical devices, is the most volatile in crisis conditions (pandemic, economic crisis, social crisis) and tends to capture a large share of the reallocated cash flows during a pandemic. The hypothesis results from the study of research carried out by some authors (5, 6, 10, 13, 17). The hypothesis supports the above defined Objective 2.

*H3*: Expenditure on healthcare services in hospitals is sensitive to the pandemic stress and generates variability in the budgetary cash-flow under the impact of the pandemic, showing an inverse trend proportional to the general trend in the evolution of realised revenues in relation to projected revenues in the healthcare system. This development has emerged from a study of the literature (1, 14–16, 18). The hypothesis is in line with Objective 3 of this research.

*H4:* Expenditure on national healthcare programmes committed to long-term strategies by policy-makers becomes volatile in a pandemic context, constituting a significant source of decreasing final allocations relative to the initial strategically committed allocations. This hypothesis is confirmed by the results of research carried out by some authors (1, 4, 5, 24–34). This hypothesis supports Objective 4 of the research.

The working hypotheses were tested using an econometric linear regression model based on the least square's method, a model based on the definition of the financing function based on the variables in Table 1. IBM-SPSS 25 statistical software was used to define the model and to test the homogeneity of the data and their statistical relevance to the phenomenon under analysis.

**Table 1:** Description of the specific indicators related to 2010-2020 period.

**Description**	**Abbreviation**	**How to calculate the indicator**	**Hint**
Dynamics of the budget revenues in the healthcare sector (%)	TIM	(realised^*^)/ (estimated^**^)	↑
Dynamics of the healthcare budget revenue (%)	TIMH	(realised^*^)/ (estimated^**^)	↑
Dynamics of the budget revenue from social assistance (%)	TIMENS	(realised^*^)/ (estimated^**^)	↑
Dynamics of the budgetary expenditure in the healthcare sector (%)	TEM	(realised^*^)/ (estimated^**^)	↑
Dynamics of the healthcare budget expenditure (%)	TEMH	(realised^*^)/ (estimated^**^)	↑
Dynamics of the budgetary expenditure in social assistance (%)	TEMENS	(realised^*^)/ (estimated^**^)	↓
Dynamics of the expenditure on materials and services in the healthcare sector compared to total expenditure (%)	TEMMATSERV	(realised^*^)/ (total realised^*^)	↑
Dynamics of the expenditure on pharmaceuticals, specific healthcare materials and medical devices in the medical sector compared to total expenditure (%)	TEMPHARMA	(realised^*^)/(total realised^*^)	↑
Dynamics of the expenditure in the healthcare sector on medical services in healthcare care facilities with beds compared to total expenditure (%)	TEMHOSP	(realised^*^)/(total realised^*^)	↓
Coverage of the healthcare programmes out of total expenditure in the healthcare sector (Drugs for high-risk chronic diseases used in the national curative programmes) (%)	TEMHDRUGSPRG	(realised^*^)/(total realised^*^)	↑
Coverage of the healthcare programmes out of total expenditure in the healthcare sector (Specific healthcare materials used in national curative programmes/total expenditure) (%)	TEMHMATPRG	(realised^*^)/(total realised^*^)	↑

* *Estimated is the planned value at the beginning of the fiscal year*.

** *Realized is the achieved value at the end of the fiscal year*.

The research population is represented by the insured population on the lists of family doctors in 2021 (16.420 million persons of the total population of 19.587 million inhabitants −83.83%) (https://cnas.ro/wp-content/uploads/2022/05/Raport-CNAS-2021-final-27-aprilie-2022.pdf, p.173). As a result, the entire population of Romania is the subject of this study as a beneficiary of health services. This is because, in Romania, even people without health insurance benefit from emergency health care. The basic foundation for the research is methodological (study of budget projections and achievements in the public health sector through statistical methods, database consolidation, classification, trend assessment, econometric modelling), empirical (study of literature), organisational (study of public health regulations and strategies issued by the Ministry of Health, Eurostat and OECD reports). The used scientific methods are of a statistical nature (development of descriptive statistics, development, validation, testing of models, development of matrices, graphical methods, and trend analysis). All these methods are used to substantiate the working hypotheses and achieve the research objectives. The scientific criteria for the selection of the methods are based on their suitability and the scientific experience that the authors have acquired over time studying and working within entities of the public health system in Romania, as well as on the basis of opportunity analyses carried out within certain research projects.

The selected variables for modelling ensure the comparability of the predicted values. On the other hand, the econometric approach takes into account the specific inputs of healthcare system financing as a lever to identify vulnerabilities in dynamics and exposures to risk factors both in terms of risk insurance elements (financing) and in terms of forecasting elements (continued financing).

The collected information resulted in a database which was analysed in terms of historical dynamics (2010–2020) as well as in terms of planned income and expenditure rates. The structure of the database has been succinctly schematized by chapters of interest and dynamic analysis indicators (see Table 1).

Using IBM- SPSS 25 software, we projected descriptive statistics over two time periods, 2010–2019 and 2019–2020. Descriptive statistics were carried out in relation to the dependent variable TIM in order to follow the evolution of the indicators. The comparison of the two statistics was made by projecting the trend indices, means and standard deviations for the indicators in Table 1(see Table 2).

**Table 2:** Descriptive statistics.

**Indicator name**	**Mean 1**	**Std. deviation 1**	**N 1**	**Mean 2**	**Std. deviation 2**	**N 2**	**Mean 2/ Mean 1**	**Std. deviation 2/ Std. deviation 1**
TIM	105.3%	4.2%	10 (2010-2019)	108.9%	2.8%	2 (2019-2020)	103.5%	65.0%
TIMH	102.5%	5.7%		105.2%	1.5%		102.6%	27.0%
TIMENS	118.8%	32.9%		200.4%	32.5%		168.8%	98.8%
TEM	105.2%	4.5%		109.2%	2.8%		103.8%	63.3%
TEMH	104.8%	5.0%		105.3%	1.4%		100.5%	28.0%
TEMENS	112.3%	26.9%		208.9%	44.5%		186.1%	165.5%
TEMMATSERV	80.4%	10.6%		61.7%	3.1%		76.8%	29.4%
TEMPHARMA	32.4%	7.6%		26.0%	3.4%		80.3%	44.4%
TEMHOSP	35.3%	6.0%		24.1%	0.7%		68.3%	11.2%
TEMHDRUGSPRG	9.9%	1.5%		9.4%	0.2%		95.2%	11.9%
TEMHMATPRG	0.9%	0.4%		1.1%	0.0%		121.8%	9.3%

Table 2 shows that the change in the dependent variable in the two pandemic years (2019-2020) has experienced an increase in dynamics on average by 3%. The same effect is observed for the dynamics of health budget revenues (2.6%), which offset the increase in health sector expenditure by 3.8%. It is noted that for the social security budget the dynamics is accelerated, registering an increase of 68% compared to the dynamics of the period intended to cover expenditure in this sector which increased by 86.1%. This resulted in an accelerated deficit in favour of expenditure of 18%. In contrast, the dynamics of expenditure on materials and services in the healthcare sector compared to total expenditure decreased compared to the average of the pre-pandemic years by 23.2%, which confirms that the budget deficit has been adjusted by policies of financial relocation of expenditure in order to cover the need to finance disease control measures.

These developments were also recorded for the other categories of expenditure, namely expenditure on pharmaceuticals, specific healthcare materials and medical devices in the medical sector compared to total expenditure (negative dynamic of 19.7%) or expenditure in the healthcare sector on medical services in healthcare care facilities with beds compared to total expenditure (negative dynamic of 31.7%). During the pandemic, the indicator Coverage of the healthcare programmes out of total expenditure in the healthcare sector (Specific healthcare materials used in national curative programmes/total expenditure) increased by 21.8%.

**Figure 1:**
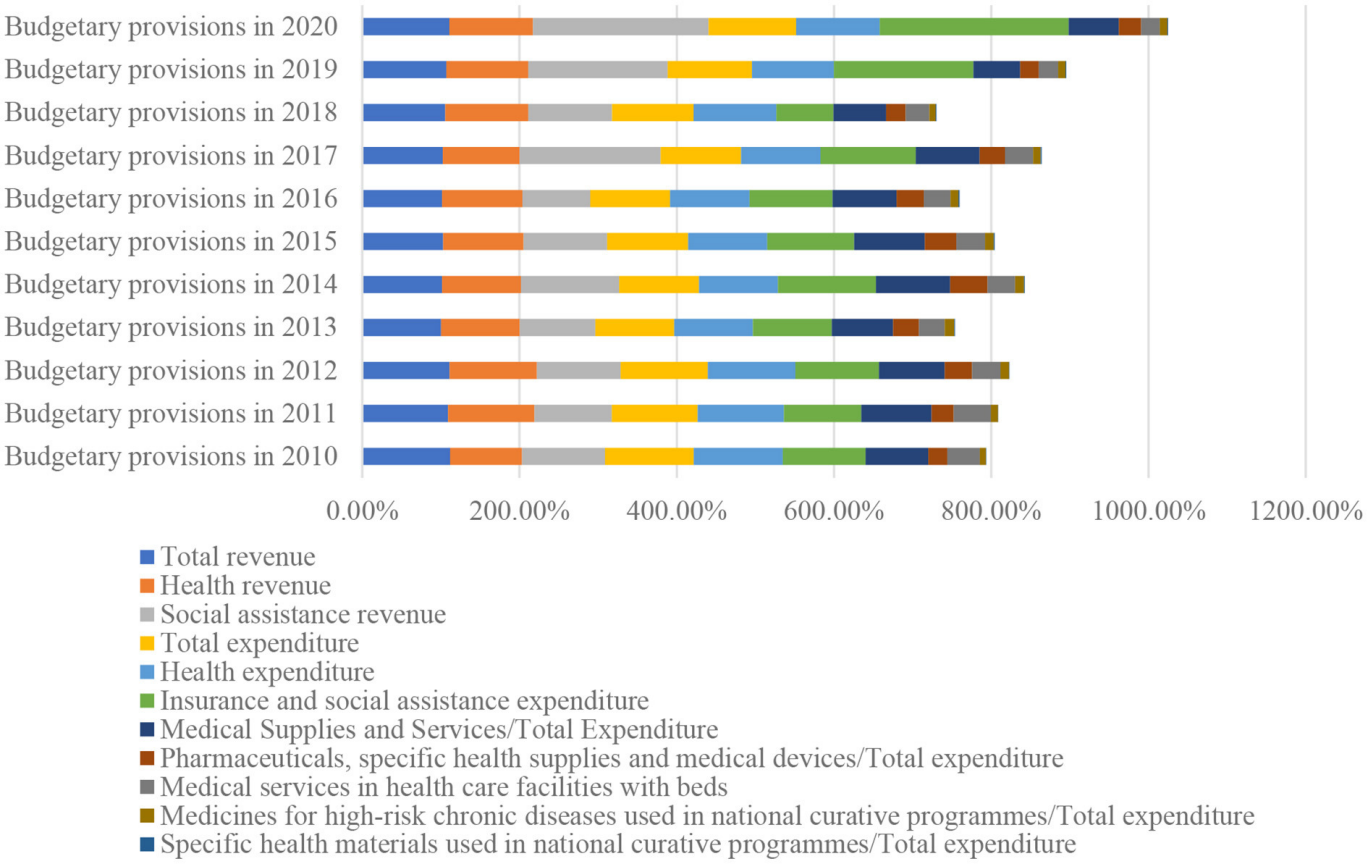
Hierarchy chart of budget provisions 2010–2020.

According to the healthcare insurance budgets (24–34), the dynamics of revenue and expenditure in the health sector are shown in Figure 1.

From Figure 1, it can be seen that the most stable dynamics of budgetary provisions over the period 2010–2020 were realised for total revenues and revenues related to the health sector. The most dynamic are the social incomes, which had the highest growth in 2014 (24.9%), 2017 (79.36%), 2019 (77.42%) and 2020 (123.44% net growth).

As far as expenditure is concerned, the amount of total expenditure followed a relatively consistent trend, demonstrating that at the level of financial policy, the revenues collected covered the financing needs in the health sector, with a more accelerated increase in expenditure than in revenues in 2010 (by 21.7%, this year revenues decreased compared to the previous year) and 2017.

The Insurance and social assistance expenditure sector represents the point of vulnerability in terms of the structure of budgetary provisions, marking the largest differences between revenue collected and expenditure incurred, as follows:

- in 2013, revenues decreased compared to the previous year by 4%, expenses remained constant (100.38% compared to the previous year);
- in 2015, revenues increased by 6% and expenses by 10%;
- in 2016, revenues decreased by 13.64% and expenses increased by 5.59%;
- in 2017, revenues increased by 79.36% and expenses increased by 21.06%;
- in 2018, revenues increased by 6.69% and expenses decreased by 27.32%;
- in 2020, revenues increased by 123.44% and expenses increased by 140.4%.

**Figure 2:**
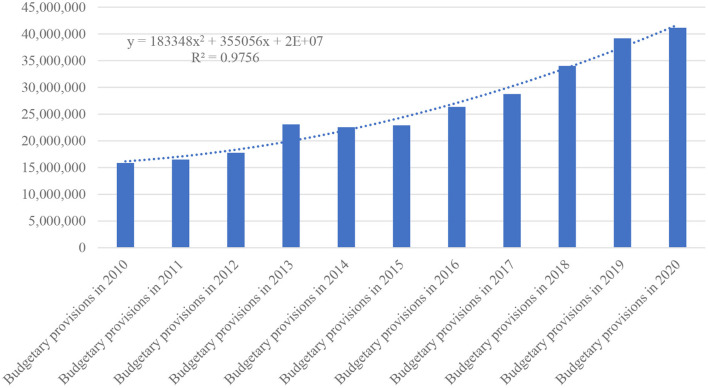
Healthcare insurance budgets provisions 2010–2020.

According to the healthcare insurance budgets from Figure 2, the evolution of collected revenues over the period 2010–2020 is an upward trend defined by the polynomial trend equation of order 2:


$$\begin{array}{l} y\;=\;183348x^{2}\;+\;355056x\;+\;2\times 10^{7}, \end{array}$$

where: y—adjusted healthcare insurance budgets provisions in forecast year n, x—magnitude of change in healthcare insurance budgets in forecast year n in monetary units.

The equation shows an average annual accumulation of 23.5%, eroded in recent years by inflation (above the 3.3% target proposed by the EU) and exchange rate volatility.

**Figure 3:**
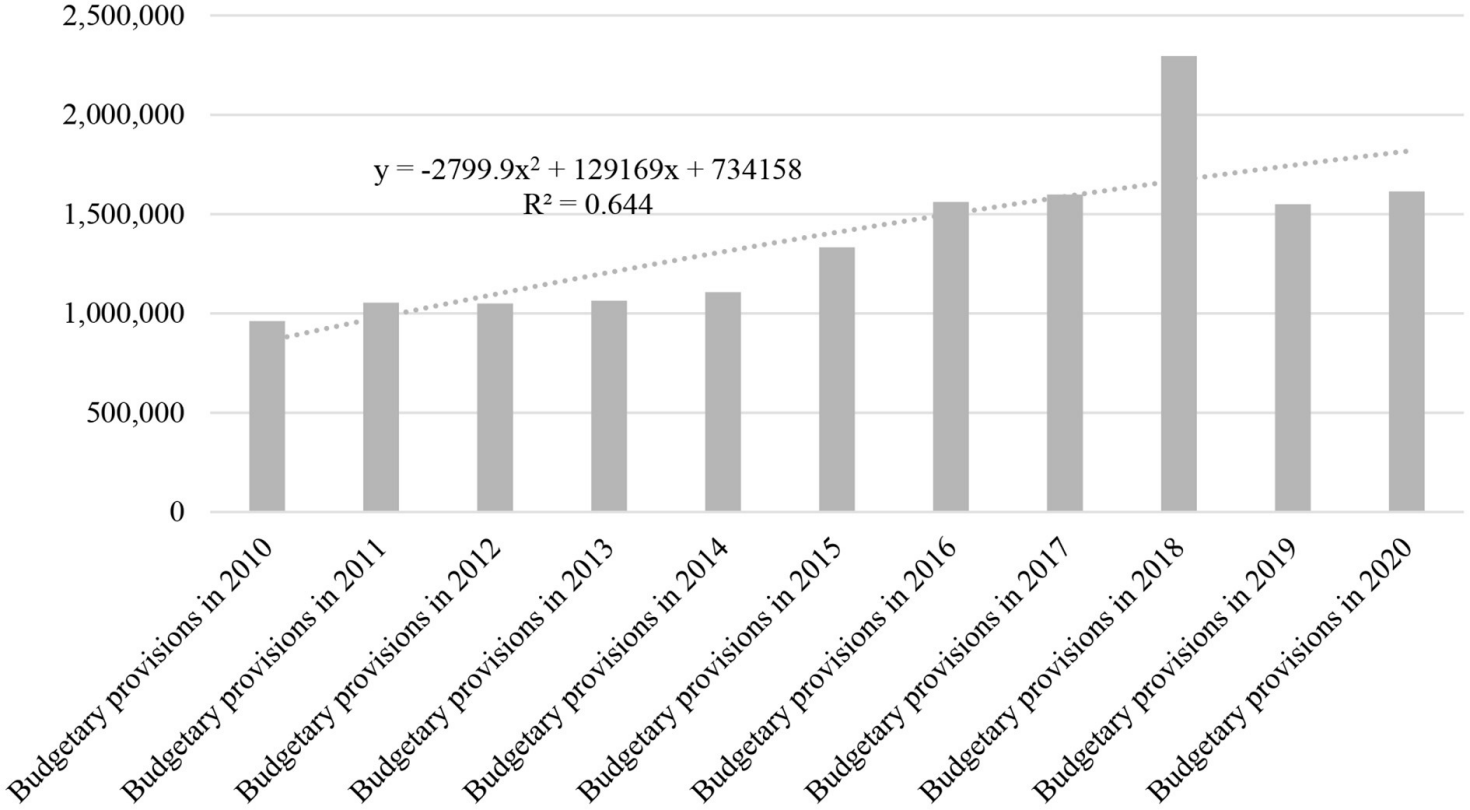
Social assistance revenue.

From the presented data in Figure 3 point of view, Social Assistance Revenue shows a trend inflection in the year 2018–2019, characterized by a low share in total revenue and a polynomial trend equation of degree 2 with negative rank 2 coefficient, expressed by the relation:


$$\begin{array}{l} y\;=\;-2799.9x^{2}\;+\;129169x\;+\;734158, \end{array}$$

where y–adjusted social assistance revenue in anul n de previziune, x–amplitudinea variatiei polinomiale a social assistance revenue in unitati monetare in anul n de previziune.

In the expenditure structure, there is an oscillating distribution of the achievements in relation to the forecast expenditure, which confirms certain structural vulnerabilities in the dynamics both in terms of exceeding the budgeted expenditure ceilings (the largest overrun was recorded in the years 2010–2012, a trend that will manifest itself again in the years 2018–2020. For the rest of the period, some stability of expenditure ceilings can be observed in terms of compliance with budgeted targets and compliance with healthcare management objectives (see Figure 2).

This is a graphical representation of the data used in the modelling, which allows to demonstrate that the data have been processed, structured, tested for homogeneity by non-parametric tests before modelling.

**Figure 4:**
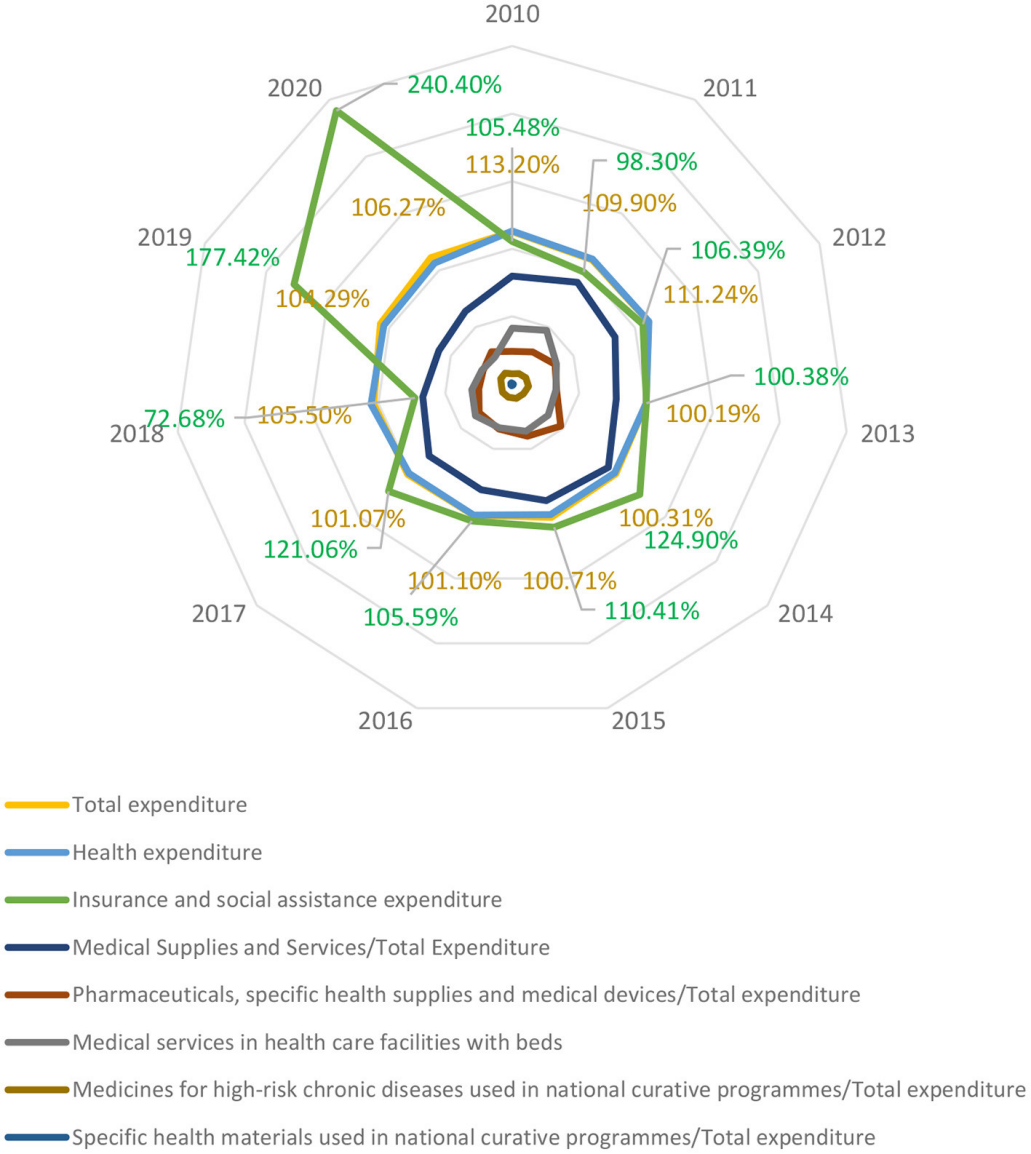
Hierarchy chart of achieved expenditure 2010–2020 under the effects of COVID-19 Pandemics.

In addition to its descriptive nature, Figure 4 shows the impact of the pandemic on the national health insurance budget and the expenditure incurred for population health protection, pandemic mitigation and disease control.

The proposed model showed that there is a significant correlation of the dependent variable, Dynamics of budget revenues in the healthcare sector (TIM), with the regressors. This variation generates a coefficient of statistical significance of almost 99.09%, no degrees of freedom on the residual variable and a Durbin—Watson coefficient of 2.25, which allows the validation of the econometric model of the form:


(1) $$\begin{array}{r} Y=\overset{n}{\underset{i=1}{\sum }}\propto_{i}\times x_{i}+\varepsilon \end{array}$$

where: Y–TIM (Dynamics of the budget revenues in the healthcare sector—dependent variable); ∝_*i*_-regression coefficients; *x*_*i*_-regressors from Table 1; ε-residual variable tending to 0; n–number of regressors (10).

The statistical tests are shown in Table 3, below:

**Table 3:** Model summary.

**Statistical test** ^a, b^	**Value**	**Result**
R	0.999	The Pearson correlation coefficient confirms the direct correlation of the regression variables of more than 99%, which generates a positive assessment of the relationship between the amounts collected and the amounts allocated to the public health budget.
R Square	99.90%	The coefficient of determination represented by the indicator R Square shows that the phenomenon, i.e., the financing of the public health system in accordance with the objectives of the allocation is 99.9% representative. This means that the dependent variable TIM is adequately represented 99.9% of the time with respect to the regressors.
Adjusted R Square	99.09%	The adjusted coefficient of determination represented by the Adjusted R Square indicator shows that the phenomenon, i.e., the financing of the public health system in accordance with the objectives of the allocation, is 99.09% representative.
Std. Error of the Estimate	0.10%	The low level of standard error of the estimator confirms the high confidence and statistical representativeness of the model.
Durbin Watson	2.25	ceeding the minimum threshold of 2 units.

a *Predictors, (Constant), TEMHMATPRG, TEMHDRUGSPRG, TEMENS, TIMH, TEMMATSERV, TEM, TIMENS, TEMPHARMA, TEMH, TEMHOSP*.

b *Dependent Variable, TIM*.

The ANOVA test reflects the homogeneity of the data by the coefficient Sig → 0, the value of the sum of squares of the regressors is 189.8, and the number of degrees of freedom allocated to the residual variable is 0. This means that all degrees of freedom are possible to be assigned to the regressors (data homogeneity) (see Table 4).

**Table 4:** ANOVA.

**ANOVA test** ^a, b^	**Sum of Squares**	**ANOVA test**	**df**	**Statistical test**	**Level**
Regression	189.805 (100%)	Regression	10/10	Mean Square	18.981
Residual	0.000 (0%)	Residual	0/0	F	0/0
Total	189.805 (100%)	Total	10/10	**Sig**.	0

a *Dependent Variable, TIM*.

b *Predictors, (Constant), TEMHMATPRG, TEMHDRUGSPRG, TEMENS, TIMH, TEMMATSERV, TEM, TIMENS, TEMPHARMA, TEMH, TEMHOSP*.

The value of the Sig coefficient allows from the one-sided critical probability test to validate the model by rejecting the null hypothesis and maintaining the alternative hypothesis with the certainty of achieving the error bounding objective within the set threshold (0.05). The result of the ANOVA test allows the assessment of the level of error attributable to the residual component as minimal, the entire value of the sum of squares being attributable to the regression function.

The results of the correlation test and the value of the unstandardized and standardized Beta coefficients, and the equation of the model are presented in Table 5.

**Table 5:** Coefficients.

**Model** ^a^	**Unstandardized coefficients**		**Standardized coefficients**	**Econometric model equation**
	**B**	**Std. Error**	**Beta**	
(Constant)	20.675	0		TIM^∧^=0.048*TIMH + 0.011*TIMENS + 0.676*TEM + 0.125*TEMH + - 0.017*TEMENS + 0.96*TEMMATSERV - 0.852*TEMPHARMA - 1.287*TEMHOSP - 0.514*TEMHDRUGSPRG - 3.33* TEMHMATPRG+20.675
TIMH	0.048	0	0.061	
TIMENS	0.011	0	0.112	
TEM	0.676	0	0.716	
TEMH	0.125	0	0.137	
TEMENS	−0.017	0	−0.184	
TEMMATSERV	0.96	0	2.465	
TEMPHARMA	−0.852	0	−1.432	
TEMHOSP	−1.287	0	−1.988	
TEMHDRUGSPRG	−0.514	0	−0.167	
TEMHMATPRG	−3.33	0	−0.278	
(Constant)	20.675	0.000	
TIMH	0.048	0.000	0.061	
TIMENS	0.011	0.000	0.112	
TEM	0.676	0.000	0.716	
TEMH	0.125	0.000	0.137	
TEMENS	−0.017	0.000	−0.184	
TEMMATSERV	0.960	0.000	2.465	
TEMPHARMA	−0.852	0.000	−1.432	
TEMHOSP	−1.287	0.000	−1.988	
TEMHDRUGSPRG	−0.514	0.000	−0.167	
TEMHMATPRG	−3.33	0.000	−0.278	

a *Dependent Variable, TIM*.

The value of the regression coefficients reflects the fact that there is an almost 1 to 1 correlation between the budget revenue allocated to healthcare and the dynamics of expenditure on materials and services in the healthcare sector compared to total expenditure. This means that there is a direct proportional relationship between allocations and available revenues.

On the other hand, we can observe a low correlation of the social assistance budget revenues in relation to the estimated income in this category, which is likely to disturb the financial projections during the pandemic. The same low correlation is found for healthcare budget revenues. Some variables have an inversely proportional distribution (TEMPHARMA—Dynamics of the expenditure on pharmaceuticals, specific healthcare materials and medical devices in the medical sector compared to total expenditure; TEMHOSP—Dynamics of the expenditure in the medical sector on medical services in healthcare units with beds compared to total expenditure). This means that the influence of the pandemic may affect these types of expenditure significantly.

Based on these observations, in the Conclusions section, we will formulate our proposals for changing public health policies in Romania.

## Results

We have presented the average evolution of the economic and financial indicators of the healthcare system financing in Table 2.

According to Table 2, Dynamics of the budget revenues in the healthcare sector is 5% on average over the study interval of 11 years. Taking into account the calculated standard dispersion, the budget adjustment capacity must be within the indicated variability limits in order to ensure efficient allocations based on the prediction function.

Relative to this indicator, the Dynamics of the healthcare budget revenue shows a more weighted trend of only 3%, which indicates that in terms of strategic planning healthcare revenue and expenditure budgets are adequately forecasted and can be affected under conditions of uncertainty by a maximum magnitude of vulnerability of 5.5%. The expenditure budget brings a strategic weighting compared to the revenue budget of 5% under a slightly higher uncertainty risk exposure (4.61% compared to 4.36%). As some authors show (4), the construction of the social healthcare system in Romania is predominantly of a public nature, an aspect that motivates the results Dynamics of the budget revenue from social assistance, which are 28% improvable and whose impact in a period of uncertainty can vary up to 44%. In the same situation is Dynamics of the expenditure on materials and services in the healthcare sector compared to total expenditure, whose exposure to risk and uncertainty is up to 46%. As far as the rest of the indicators are concerned, there is a significant need for predictability, which is a wake-up call for healthcare and social policy elements both in terms of healthcare infrastructure (construction of regional hospitals, redistribution of beds in relation to the real healthcare needs of the population) and in terms of aspects related to financing programmes for chronic diseases which represent programmes of high social interest both because of the exclusive costs of treatments and because of the major social impact on the population affected by chronic diseases.

### Expenditure on national healthcare programmes committed to long-term strategies

**Figure 5:**
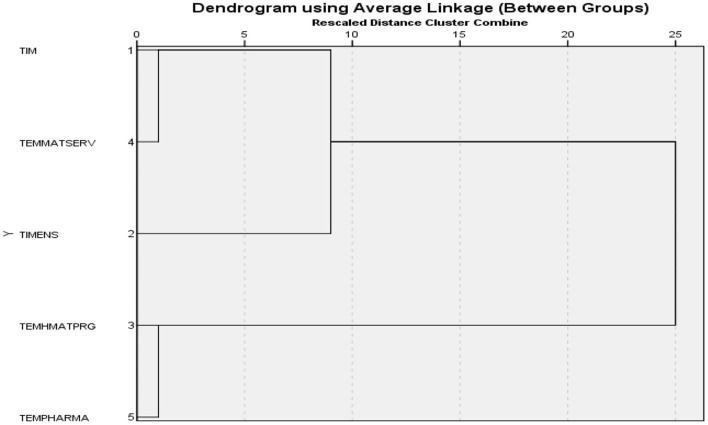
Average Linkage (Between Groups).

It can be seen that there is an average predictability of variations so that the maximum variation of the annual financial indicators is achieved in the case of the dynamics of budget expenditure in social assistance (realised vs. estimated), while the minimum variation is achieved for allocations in the healthcare programmes system, which outside the pandemic undergoes a small change in values between allocation and realisation (Figure 5).

From the presented dendrogram, an asymmetry of the frequency distributions results, and it is necessary to identify the disturbance elements that, through the proximity matrix, can be evaluated in an adequate way (see Table 6).

**Table 6:** Proximity matrix.

**Case**	**Matrix file input**				
	**TIM**	**TIMENS**	**TEMHMATPRG**	**TEMMATSERV**	**TEMPHARMA**
TIM	0.000	0.921	0.408	1.000	0.728
TIMENS	0.921	0.000	0.000	0.768	0.395
TEMHMATPRG	0.408	0.000	0.000	0.689	0.992
TEMMATSERV	1.000	0.768	0.689	0.000	0.920
TEMPHARMA	0.728	0.395	0.992	0.920	0.000

### The budgetary revenues from social assistance

The hypothesis validation is based on the Pearson correlation table (see Table 7).

**Table 7:** Pearson correlation for dependent variable.

**Variable**	**Pearson correlation**	**Variable**	**Pearson correlation**
TIM	1.000/1.000	TEMMATSERV	−0.292/−1.000
TIMH	0.266/1.000	TEMPHARMA	−0.569/−1.000
TIMENS	0.242/1.000	TEMHOSP	0.084/1.000
TEM	0.988/1.000	TEMHDRUGSPRG	−0.546/−1.000
TEMH	0.931/1.000	TEMHMATPRG	−0.580//1.000
TEMENS	0.316/1.000		

Also from Table 7 it emerges that the Pearson correlation-based volatility index of social care budget revenue is a 24% dependent on the dependent variable (healthcare budget revenue), which confirms *H1*: The budgetary revenues from social assistance, under pandemic conditions, may be affected until the trend function changes in inverse proportion to budgetary revenues in the healthcare sector in relation to Research Objective 1: Identifying budget revenue categories that react sensitively to pandemic risk.

### Spending on pharmaceuticals and specific healthcare supplies

In terms of pharmaceutical expenditures, Table 7 shows that they reach an inverse proportional correlation of 57%, which means that these expenditures under the impact of the pandemic tend to volatilize, which confirms H2: Spending on pharmaceuticals and specific healthcare supplies, including medical devices, is the most volatile in crisis conditions and tends to capture a large share of the reallocated cash flows during a pandemic. The hypothesis supports Objective 2 defined above, i.e., Identifying categories of budget expenditure that react in a sensitive way to pandemic risk.

### Expenditure on healthcare services in hospitals

The expenditure on medical services in hospitals shows an inversely proportional variation with a low correlation of 29% with the dependent variable, which demonstrates *H3*: Expenditure on healthcare services in hospitals is sensitive to the pandemic stress and generates variability in the budgetary cash-flow under the impact of the pandemic, showing an inverse trend proportional to the general trend in the evolution of realised revenues in relation to projected revenues in the healthcare system. Hypothesis 3 is consistent with Research Objective 3: Identify expenditure categories that influence budget cash-flow variability and generate budget sensitivity under pandemic conditions.

Figure 5 and Table 6 support hypothesis *H4*: Expenditure on national healthcare programmes committed to long-term strategies by policy-makers becomes volatile in a pandemic context, constituting a significant source of decreasing final allocations relative to the initial strategically committed allocations and Research Objective 4: Quantify the effects of the pandemic on long-term strategic objectives in health by forecasting the outcomes of the proposed model.

**Figure 6:**
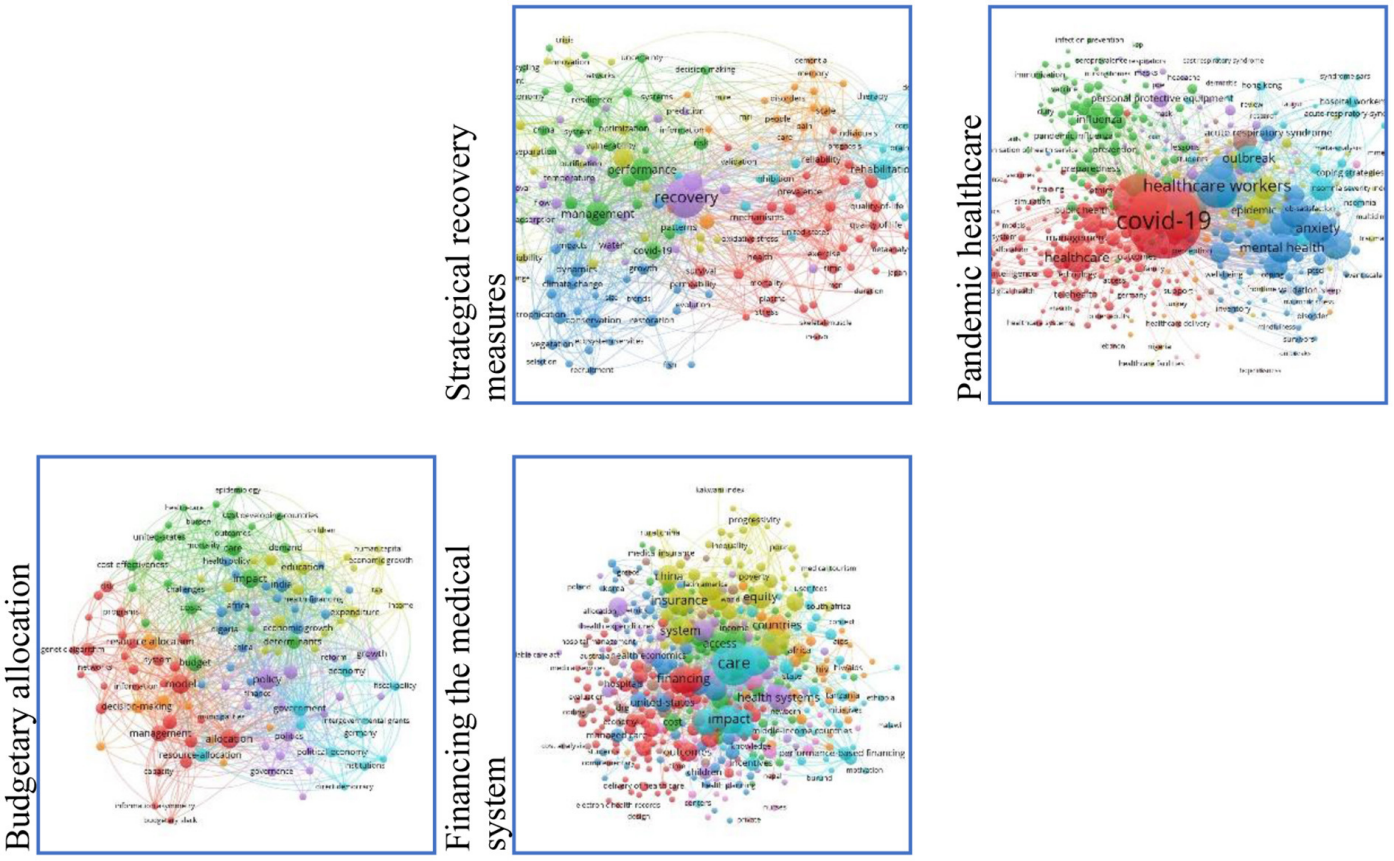
Research topics and researchers' interest.

We used the Web of Science platform and VosViewer software to query interest of other researchers in this topic. The results in Figure 6 show that the topic addressed in this scientific endeavour is of immense interest to researchers. A multitude of studies have been carried out in this regard.

## Discussion

**Figure 7:**
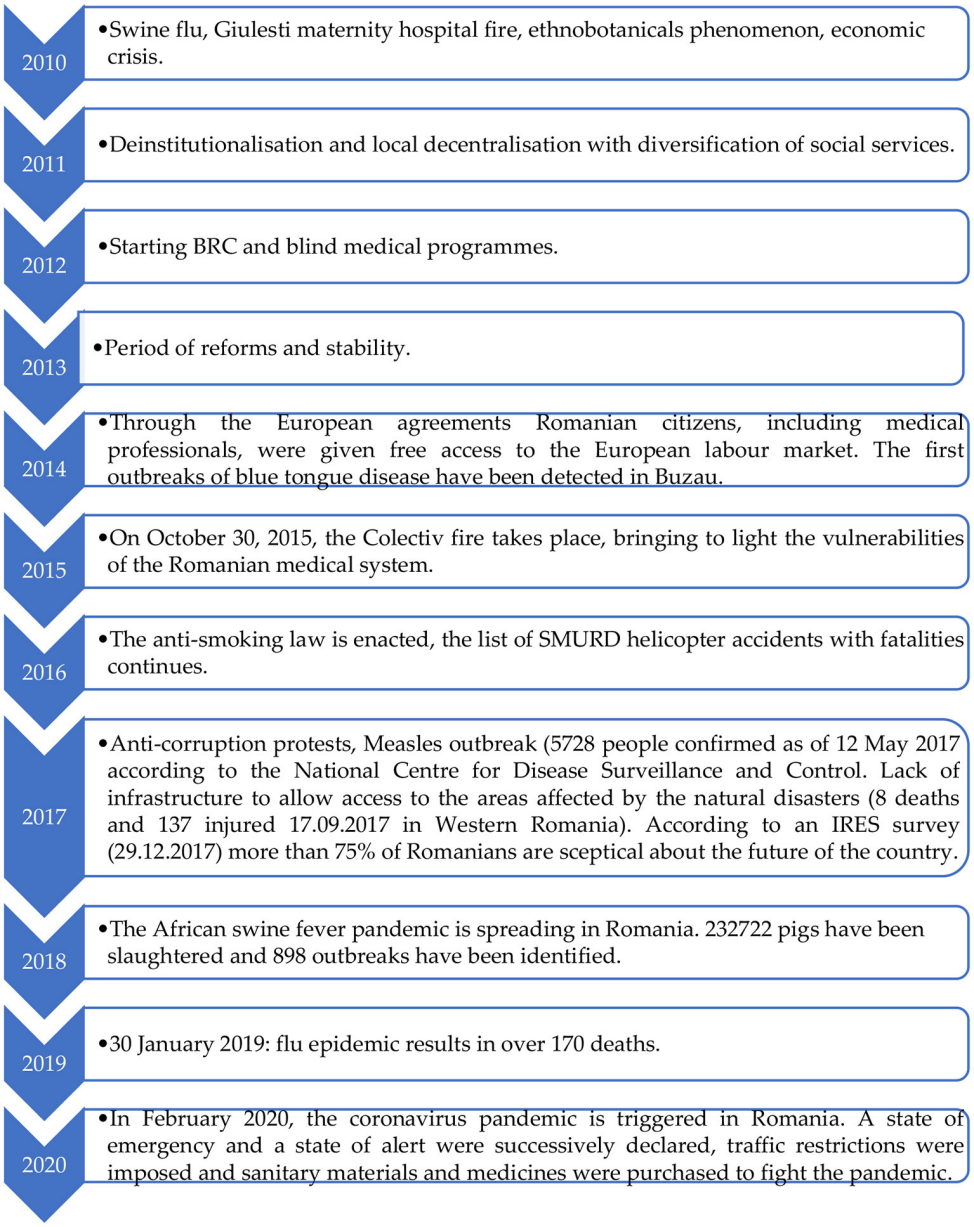
Crisis' effects on healthcare expenditure approach in Romania.

In Romania there are common features with the Member States analysed above regarding the management of the healthcare system and sources of funding. In terms of dynamics, the financing of the healthcare sector has faced certain moments of crisis which in turn have had a negative impact on the indicators analysed. The effects diagram is shown in Figure 7.

Figure 7 highlights a series of events (shocks) that have marked the health security policy in Romania and that have influenced the budgetary expenditure in the field. This approach is also supported by research carried out by (35). As a result, the allocation of funds in healthcare system has always aimed to strike a balance between problem and solution. However, the results of the allocation management strategy have generated inconsistencies that perpetuate imbalances from year to year.

The healthcare budget financing system in Romania presents particularities compared to other Member States. In France, for example, the healthcare system is centralised and has regional responsibilities. As in Romania, the French state is becoming increasingly involved in controlling healthcare expenditure. During the pandemic outbreak and afterwards, healthcare expenditure in France increased above the EU average, a situation also present in Germany (36). Germany can boast of the oldest social healthcare insurance (SHI) system in the world. However, financing the healthcare system faces administrative and financial difficulties. The healthcare financing system is dual (federal and regional). The pandemic has highlighted dysfunctions in the financing of the German healthcare system related to federal systems in coordinating and managing. Germany are the highest healthcare expenditure among EU. Moreover, extra public funding was approved in 2020 and 2021 to support the healthcare sector (37). And in the Austrian healthcare system, the state is heavily involved. The federal level of involvement is doubled by the regional level. As a result, healthcare financing is mixed. Pandemic has highlighted shortcomings in the coordination of decisions across federal and state levels in the circumstances that Austria has one of the most expensive healthcare systems in the EU (38). Spain presents a decentralised healthcare system, but one that is funded by taxes. Management of the healthcare system is provided by the state on the one hand and the regional authorities on the other. Spain's healthcare spending has increased in recent years but remains below the EU average. Starting 2020, the government approved extra injections of funds for the regions which faced to the pandemic (39). Italy has a decentralised regional structure of the healthcare system, based on tax funding. This structure continued to function during the pandemic period, even though leadership and administrative authority were transferred to government bodies. The budget allocation for financing the healthcare system is below the EU average (40). Hungary has a strongly centralised healthcare system. The Ministry of Health defines strategic direction, controlling financing, determining the benefits package and issuing and enforcing regulations. Although funding during the pandemic crisis has increased, it remains below the European average (41). Greece has a centralised public healthcare system. There are private providers, mainly to deliver primary and outpatient care and diagnostic services (42).

A step forward in the analysis is to compare the dynamics of health insurance expenditure per capita in the Member States. Eurostat data are available until 2019 (43). According to these data, Romania ranks last in every year of the period analyzed, except for 2019, when it overtook Bulgaria.

Luxembourg, Sweden and Denmark are the countries with the highest spending per capita. The difference between the level of spending in these countries and Romania ranges from 2037% in 2010 to 878% in 2019. The trend graph shows an approximation of the level of expenditure in the 2010–2019 period.

We believe that a problem-solving rebalancing of allocations could help to eliminate the synergy in health. This redistribution should take into account the impact that economic and budgetary factors have on healthcare factors and vice versa, so that at some point after successive adjustments the minimum distance between forecast and realisation or between need and financing of need can be reached.

The limitations of the study concern the relatively short period of time in relation to the size of the disturbances produced on the medical system in Romania, but also in relation to the process of changing the strategic planning in terms of medical financial management. The number of indicators may be a limitation of the study, limiting the identification of vulnerability. On the other hand, the available financial data cover only the first 9 months of the pandemic. At this moment the official healthcare budget on 2021 in its final form is not finished.

## Conclusions

The authors conducted a critical research related to the financing of the public health system in Romania, using official data that were analyzed in dynamics in order to eliminate the seasonality of the data and to quantify trends in the evolution of budget indicators related to the medical sector.

From the study, it emerged that the health system in Romania is experiencing the effects of financial policy changes, especially due to the change in the share of funding of some health programmes, which ranks Romania on one of the last places in the EU and increases the risk of managing health crises.

Contributions to the development of the field include:

- identifying the best practices to approach financial policies in the public health sector in the current conditions, where the effects of the pandemic are added to the security risks related to the current geo-political context;
- starting from the financial indicators on revenues and allocations from public sources related to the health sector, we have shown that there are dysfunctions especially in the expenditure segment, which tend to exceed public programs and adapt in crisis situations to specific needs determined by social security interests or public financial interests specific to intra-budgetary allocations;
- the proposed model demonstrated that revenue and expenditure indicators are in a direct correlation represented by the interests promoted at national level in terms of budget allocations. In particular, some financial sectors such as insurance and social assistance expenditure show atypical behaviour generated by the impact of the international context and the economic vulnerabilities it induces;
- the dendogram presented shows that there are connections between the categories of financial indicators, the most representative of which are at the level of the dynamics of the budget allocated to social assistance expenditure;
- the results obtained place the presented model in a generally unstable context that should be improved by consistent financial policies, based on reallocations according to medium-term strategies and not according to specific interests, as it is currently done.

The study focuses on the impact of financial policy changes in the healthcare sector on the insured population in Romania, i.e., 83.83% of the total Romanian population.

The hypotheses of the study, validated by statistical calculations, showed that: the budgetary revenues from social assistance, under pandemic conditions, may be affected until the trend function changes in inverse proportion to budgetary revenues in the healthcare sector; spending on pharmaceuticals and specific healthcare supplies, including medical devices, is the most volatile in crisis conditions (pandemic, economic crisis, social crisis) and tends to capture a large share of the reallocated cash flows during a pandemic; expenditure on healthcare services in hospitals is sensitive to the pandemic stress and generates variability in the budgetary cash-flow under the impact of the pandemic, showing an inverse trend proportional to the general trend in the evolution of realised revenues in relation to projected revenues in the healthcare system; and expenditure on national healthcare programmes committed to long-term strategies by policy-makers becomes volatile in a pandemic context, constituting a significant source of decreasing final allocations relative to the initial strategically committed allocations.

In relation to the objectives of the research, it emerges that, under the impact of pandemic stress, measures to improve healthcare management, increase performance and streamline financial allocation are vulnerable. Moreover, they cannot counteract the effects that the pandemic has on the healthcare of the population as reflected in the morbidity and mortality indicators collected during the pandemic.

In this regard, a rethinking of the strategic healthcare management, a better planning of the procurement of medicines and healthcare supplies, a rethinking of the partnerships with the European Commission and other global entities that can effectively improve the impact of the pandemic on the healthcare status of the population, a rebalancing of the demand-supply balance in healthcare and a maintenance of the strategic programmes, according to the objectives assumed in the planning, given that these programmes protect categories of people already medically affected.

As a result of the analysis, we can state that the working hypotheses have been fully confirmed, demonstrating that the health system in Romania is vulnerable in terms of funding and exposure to health risk (pandemic), with multiple events due to lack of funding and management vision (fires, sentinel events, adverse events, etc.).

This research is addressed in particular to bodies with decision-making abilities in the healthcare sector but also to other political decision-makers and proposes, after identifying the vulnerabilities related to financial management in health, some strategic directions to be analysed by them, directions that will prevent future disruptions produced in the period 2020-2021 by the pandemic.

The analysis carried out by the authors highlighted the weaknesses of the public health system in Romania consisting of underfunding, low acquis communitaire, bureaucracy, reactive approach to the problems in the field, distorted distribution of funds in relation to previous projections made (dynamics with high dispersion especially in the area of budget execution) and lack of predictability of sustainable measures in case of risk events such as pandemic.

In view of these elements, the proposals for improving public health policies are based on ensuring transparency and predictability of budget execution, reallocation of funds based on cost-benefit analysis, development of aquis communitaire (import of know-how), real and effective implementation of digitalisation, the implementation of efficient methods for calculating the average cost of diagnosis and monitoring the elements that lead to overconsumption, a new approach to the financing policy in relation to the National Social Insurance House (CNAS in Romanian), and last but not least, increasing the organizational capacity by providing an adequate information system capable of connecting all health units, a system that ensures both feed-back and feed-in of information.

According to the new financial perspective at EU level, Romania has defined and proposed for implementation (44) the Health Operational Programme 2021–2027 (45). The official document states that health expenditure in Romania is the lowest in the EU, representing 5% of Romania's GDP, i.e., 1029 euros per capita. At EU level, the average values of these indicators are much higher, respectively 9.8% and 2,884 euros.

In order to achieve the goals of this programme, the financial support comes from the European Regional Development Fund (ERDF), the European Social Fund+ (ESF+) and National Co-financing—State Budget (CN-BS). The concrete measures aim at two major objectives: Investments for the construction of re-regional hospitals and new hospital infrastructures with major territorial impact and Improving the effectiveness of emergency medical services.

There are also specific objectives related to:

- increasing access to primary health care, community and outpatient services;
- improving accessibility and effectiveness of rehabilitation/recovery services, palliative care services, long-term care services;
- increasing the effectiveness of the health sector through investment in infrastructure and services;
- improving the effectiveness and efficiency of healthcare services through investment in research and digitisation of the healthcare system;
- increasing the use of modern and innovative methods of investigation, intervention and treatment.
- In order to achieve these objectives, we recommend the following measures to improve public health policies:
- increasing the transparency of decision-making regarding the use of public funds in health and introducing budget efficiency indicators by comparing the achievement of planned objectives with the objectives actually achieved at the end of the calendar year in quantitative and qualitative terms;
- reducing bureaucracy, implementing digital systems for analysis and adoption of public health policies;
- proactively addressing health issues;
- improving the time management of decision making and implementation of public health policies;
- application of the prioritisation matrix of problems requiring public health policies and allocation of priority funds according to the results of the prioritisation matrix;
- moving to stage 3 of accreditation of hospitals in Romania after completion of stage 2 and accreditation of the remaining 400 unaccredited hospitals, with the issuance of national quality standards (3rd edition) in line with those promoted at European level; increasing the acquis communautaire.

The authors plan to extend this research in a future study by increasing the number of indicators used and extending the statistical period of analysis to take into account the trend at European not national level and the new challenges due to the pandemic and the war in Crimea.

The data used in the study are the data reported for the period 2010-2020 by National Health Insurance House in Romania, through published expert reports.

## Data availability statement

Publicly available datasets were analyzed in this study. This data can be found here: https://cnas.ro/rapoarte-de-activitate.

## Author contributions

Conceptualization: VA and RI. Methodology: RI, MZ, and VA. Validation and formal analysis: CM and MZ. Investigation: RI and CM. Resources and visualization: VA. Data curation and supervision: RI. Writing—original draft preparation: RI, AM, and MZ. Writing—review and editing: MZ, CM, and RI. All authors contributed to the article and approved the submitted version.

## Conflict of interest

The authors declare that the research was conducted in the absence of any commercial or financial relationships that could be construed as a potential conflict of interest.

## Publisher's note

All claims expressed in this article are solely those of the authors and do not necessarily represent those of their affiliated organizations, or those of the publisher, the editors and the reviewers. Any product that may be evaluated in this article, or claim that may be made by its manufacturer, is not guaranteed or endorsed by the publisher.

## Abbreviations

EU: European Union
OECD: The Organisation for Economic Co-operation and Development
FNUASS: Single National Health Insurance Fund
CNAS: National Health Insurance House
NHIF: National Health Insurance Fund
DRG: Hospital care provider payment system
TIM: Dynamics of the budget revenues in the health sector
TIMH: Dynamics of the health budget revenue
TIMENS: Dynamics of the budget revenue from social assistance
TEM: Dynamics of the budgetary expenditure in the health sector
TEMH: Dynamics of the health budget expenditure
TEMENS: Dynamics of the budgetary expenditure in social assistance
TEMMATSERV: Dynamics of the expenditure on materials and services in the health sector compared to total expenditure
TEMPHARMA, Dynamics of the expenditure on pharmaceuticals: specific health materials and medical devices in the medical sector compared to total expenditure
TEMHOSP: Dynamics of the expenditure in the health sector on medical services in health care facilities with beds compared to total expenditure
TEMHDRUGSPRG: Coverage of the health programmes out of total expenditure in the health sector (Drugs for high-risk chronic diseases used in the national curative programmes)
TEMHMATPRG: Coverage of the health programmes out of total expenditure in the health sector (Specific health materials used in national curative programmes/total expenditure)
SPSS: Statistical Product and Service Solutions
GDP: Gross Domestic Product.
